# Characterisation of multidrug-resistant, avian pathogenic-like *Escherichia coli* O78 from dairy-bred calves with atypical colisepticaemia

**DOI:** 10.1371/journal.pone.0359646

**Published:** 2026-10-09

**Authors:** Jolinda Pollock, Geoffrey Foster, Jordan Maliska, Katrina Henderson, Colin Mason, Cici Corbett, Gavin K. Paterson

**Affiliations:** 1 SRUC Veterinary Services, Edinburgh, United Kingdom; 2 Royal (Dick) School of Veterinary Studies and the Roslin Institute, University of Edinburgh, Edinburgh, United Kingdom; Federal University Dutse, NIGERIA

## Abstract

The objective of this work was to identify and characterise the causative agent(s) of an atypical septicaemic syndrome in 3- to 6-month-old calves. Nine cattle from three Scottish dairy farms were subject to post-mortem in 2020 and 2021, and tissue samples were screened via routine bacteriology. *Escherichia coli* was isolated from all sampled tissues and a representative isolate from each calf was taken forward for whole-genome sequencing for strain typing, identification of putative virulence factors and antimicrobial resistance genes, and assessment of phylogenetic relationships with other sequenced strains. The nine *E. coli* isolates were highly-related, being separated by a maximum of 5 single nucleotide polymorphisms. The isolates were serotyped *in silico* as O78, H9 and were multilocus sequence typed as ST23. Further analysis revealed 13 virulence genes, with the most frequent being associated with serum survival, cytotoxic protein and haemolysin production, and iron transport. These strains encode a novel *fimH* variant – designated *fimH*-1571 – which has a mutation potentially linked to increased adherence and invasion. Phylogeny and virulence gene profile indicate an avian pathogenic-like *E*. *coli* within a poultry lineage, however there were no poultry units in the vicinity of these farms. Ampicillin, amoxicillin, streptomycin and tetracycline resistance was predicted from their genome sequences, which agreed with phenotypic antimicrobial susceptibility testing. In conclusion, while not demonstrated here, these data are consistent with a transmission event between cattle units (or a common source) and suggest the possible emergence of virulent avian pathogenic-like *E*. *coli* in cattle for which future surveillance is merited.

## Introduction

*Escherichia coli* is one of the most common bacterial causes of diarrhoea and septicaemia in calves [1], with enterotoxigenic *E*. *coli* (ETEC) and attaching and effacing *E*. *coli* (AEEC) being among the principal pathotypes associated with calf diarrhoeal disease [2]. Infection may remain localised to the intestine causing severe diarrhoea but can also manifest as a systemic infection known as colisepticaemia. Colisepticaemia is predominantly recognised in neonatal calves, often in the first few days of life, with susceptibility influenced by factors such as the inadequate transfer of maternal antibodies and exposure to pathogenic *E*. *coli* [3–5].

In this study, we sampled nine calves at post-mortem from three Scottish dairy units that presented with a severe septicaemic syndrome. The presenting clinical signs in the majority (six out of nine) calves were neurologically abnormal behaviour progressing rapidly to death or seizures and consequential euthanasia. Pyrexia and recumbency were reported in some and multiple swollen joints were noted in three cases. Bacterial culture revealed the presence of *E. coli* in each of the sampled tissues, which is an unusual clinical finding in 3–6-month-old calves. Furthermore, these isolates were phenotypically indistinguishable, suggestive of a link between the cases. Therefore, nine *E. coli* isolates, one from each individual calf, were subject to whole-genome sequencing for strain typing, and also to provide information on the relatedness of the study strains, virulence and antimicrobial resistance determinants, and phylogenetic relationships with other publicly available *E. coli* genomes. The results showing that all isolates from the nine individual calves and the three premises were highly-related and shared an avian pathogenic-like *Escherichia coli* genotype.

## Methods

### Cattle unit descriptions and clinical findings

Three cattle units located in Dumfries and Galloway, Scotland, United Kingdom were studied, termed Units A, B and C. These units were within a 10-mile radius with each being served by a different veterinary practice but no investigations of other links between the premises were possible. The nine studied calves were between 3 and 6 months of age and the disease presented as pericarditis, joint ill, meningitis, septicaemia and endotoxemia (Table 1). Tissue samples were selected at post mortem for bacterial culture.

**Table 1:** Source and characteristics of representative calf colisepticaemia isolates genome sequenced in this study.

Isolate	Unit	Calf Age in months	Sampling Date	Source of isolate	Phenotypic resistance	AMR genes
C468983	A	4	August 2020	Spleen (+++), *E*. *coli* also isolated from brain (+++).	ampicillin, amoxicillin-clavulanic acid streptomycin, and tetracycline	*bla*_TEM-1B_, *aph*(3”)-*Ib*, aph(6)-*Id*, *tet*(B)
C468866	A	3	August 2020	Brain (+++), *E*. *coli* also isolated from cerebrospinal fluid (+++).	ampicillin, amoxicillin-clavulanic acid streptomycin, and tetracycline	*bla*_TEM-1B_, *aph*(3”)-*Ib*, aph(6)-*Id*, *tet*(B)
C468904	A	3	August 2020	Brain (++), *E*. *coli* also isolated from lung (++), liver (++), spleen (+++) and synovial fluid (++).	ampicillin, amoxicillin-clavulanic acid streptomycin, and tetracycline	*bla*_TEM-1B_, *aph*(3”)-*Ib*, aph(6)-*Id*, *tet*(B)
C469032	A	6	September 2020	Lung (+++), *E*. *coli* also isolated from brain (+++).	ampicillin, amoxicillin-clavulanic acid streptomycin, and tetracycline	*bla*_TEM-1B_, *aph*(3”)-*Ib*, aph(6)-*Id*, *tet*(B)
C469075	A	4	September 2020	Brain (+++), *E*. *coli* also isolated from lung (+++).	ampicillin, amoxicillin-clavulanic acid streptomycin, and tetracycline	*bla*_TEM-1B_, *aph*(3”)-*Ib*, aph(6)-*Id*, *tet*(B)
C469009	B	5	September 2020	Lung (+++)	ampicillin, amoxicillin-clavulanic acid streptomycin, and tetracycline	*bla*_TEM-1B_, *aph*(3”)-*Ib*, aph(6)-*Id*, *tet*(B)
C469142/1	B	3	September 2020	Brain (+++), *E*. *coli* also isolated from spleen (+++) and front joints (+++).	ampicillin, amoxicillin-clavulanic acid streptomycin, and tetracycline	*bla*_TEM-1B_, *aph*(3”)-*Ib*, aph(6)-*Id*, *tet*(B)
C469142/2	B	3	September 2020	Brain (+), *E*. *coli* also isolated from spleen (+) and rear joint (++).	ampicillin, amoxicillin-clavulanic acid streptomycin, and tetracycline	*bla*_TEM-1B_, *aph*(3”)-*Ib*, aph(6)-*Id*, *tet*(B)
C470773	C	4	October 2021	Pericardium (+++), *E*. *coli* also isolated from liver (+++), lung (+++) and hock (+++).	ampicillin, amoxicillin-clavulanic acid streptomycin, and tetracycline	*bla*_TEM-1B_, *aph*(3”)-*Ib*, aph(6)-*Id*, *tet*(B)

A single isolate was taken from each affected calve. All units are in Dumfries and Galloway, Scotland, UK. All nine genome sequenced isolates belonged to serotype 078, sequence type 23, were K99-negative, encoded a novel *fimH* variant, 1571 and produced the same API 20E numerical profile, 5144552. In addition, all isolates were sensitive to ertapenem, enrofloxacin, cefotaxime, trimethoprim-sulphamethoxazole, florfenicol, apramycin and ceftiofur. Description of growth, based on extent of growth after sequential dilution by streaking, defined in brackets as + light (10–100 colony-forming units (cfu)), ++ moderate (100–1000 cfu) and +++ heavy (1000 + cfu).

### Routine bacteriology, antimicrobial susceptibility testing and K99 ELISA

Samples for bacteriology were cultured on Columbia sheep blood agar (Oxoid, Basingstoke, United Kingdom) incubated at 37°C in air plus 5% CO_2_ and MacConkey agar without salt (Oxoid) incubated aerobically at 37°C. Selective cultures on all faecal samples were also performed for *Salmonella* species on Brilliant Green Agar and through Selenite F Broth enrichment (Oxoid). Clinically significant isolates were identified by Matrix Assisted Laser Desorption Ionisation-Time of Flight Mass Spectrometry (MALDI-TOF) and API 20E strips (BioMerieux, Basingstoke, United Kingdom). Antimicrobial susceptibility testing was performed by disc diffusion according to the European Committee of Antimicrobial Susceptibility Testing [6], Clinical and Laboratory Standards Institute [7] and Comité de l’antibiogramme de la Société Française de Microbiologie breakpoints [8]. The tested antimicrobials were ampicillin, streptomycin, amoxicillin-clavulanic acid, ertapenem, enrofloxacin, tetracycline, cefotaxime, trimethoprim-sulphamethoxazole, florfenicol, spectinomycin, apramycin and ceftiofur with the interpretive criteria used shown in Table S1 in S1 File. The presence/absence of *E.coli* K99 was measured by ELISA using a Pathasure Enteritis 4 kit (Biovet, United States), according to the kit instructions, using a single bacterial colony immersed in wash buffer.

### Whole genome sequencing and bioinformatics

Representative post mortem *E. coli* isolates from each of the nine affected calves (Unit A: n = 5, Unit B: n = 3, Unit C: n = 1) were submitted for whole genome sequencing (MicrobesNG, Birmingham, United Kingdom) (Table 1). DNA extraction and sequencing using the Illumina HiSeq platform were carried out as per the company’s standardised protocol. Briefly, genomic DNA was extracted using Solid Phase Reversible Immobilization beads and genomic DNA libraries were prepared using the Nextera XT Library Prep Kit (Illumina) following the manufacturer’s protocol, with the following modifications: input DNA increased 2-fold, and PCR elongation time was increased to 45 s. Libraries were then sequenced with Illumina sequencers (HiSeq/NovaSeq) using a 250 bp paired-end protocol. Adapters were removed from the generated reads using Trimmomatic 0.30 [9] using a sliding window quality cut-off of Q15. *De novo* assembly was performed on samples using SPAdes version 3.7 [10] and contigs annotated using Prokka 1.11 [11].

### Sequence analysis

A core genome SNP-based phylogeny was generated using the refMapper v3 pipeline in Enterobase v.1.1.4 [12] with APEC O78 as the reference (accession GCF_000332755). This was comprised of the nine sequenced *E*. *coli* from this study and a further 191 ST23 *E*. *coli* from Enterobase (Table S1 in S1 File), selected to represent a range of geographical locations and host species. There was a total of 35365 variant sites. Using Enterobase v.1.1.4 the resulting SNP alignment was used to infer a maximum likelihood phylogeny using RAxML v8.2.4 under the ASC_GTRCAT model with ascertainment bias correction for SNP-only data. The resulting tree was visualised using GrapeTree [13]. Multilocus sequence typing (MLST), antimicrobial resistance genes, serotype, *fimH* type and virulence-associated genes, were extracted from genome sequences using MLST 2.0 [14], ResFinder 4.1, SerotypeFinder 2.0 [15], FimTyper 1.0 [16] and VirulenceFinder 2.0 [17,18], respectively, which are available on the Center for Genomic Epidemiology website (http://genomicepidemiology.org/).

### Nucleotide accessions

Sequencing reads from this study have been deposited with GenBank under the following accessions: C468866, SRR15367468; C468904, SRR15367469; C469075, SRR15367470; C469009, SRR15367471; C469142/1, SRR15367472; C470773, SRR19881318; C469032/1, SRR19881319; C468983, SRR19881320; C469142/2, SRR19881322. See Table S1 in S1 File for details of Enterobase entries for these nine study isolates.

### Animal ethics

Samples were collected with informed consent as part of routine veterinary diagnostic investigations, by qualified veterinarians, with no additional procedures performed for research purposes. The work therefore, constituted routine veterinary practice/clinical veterinary research under RCVS guidance.

## Results

### Bacteriology

In total, 24 post mortem samples from nine affected calves were taken for bacteriology, these comprised of seven brain, five lung, four spleen, three joint, two liver, one cerebrospinal fluid, one synovial fluid and one pericardial sample (Table 1). All faecal samples were tested for *Salmonella* species, on both direct and enriched cultures and were negative. In addition, all samples were plated onto MacConkey agar and none yielded non-lactose fermenting colones. However, pure, or dominant growths of non-haemolytic *E. coli* was isolated from each of the 24 sampled tissues, in most cases as moderate to heavy growth. Isolates were identified as *E*. *coli* by MALDI-TOF with 21 of these also tested by API 20E, returning the same numerical profile of 5144552, indicating phenotypic similarity, consistent with sucrose-negative *E*. *coli*. Nine isolates, one from each individual calf were selected for further study (Table 1 and S1 Table in S1 File). All nine of these isolates shared the same antimicrobial resistance profile, displaying resistance to ampicillin, amoxicillin-clavulanic acid, streptomycin, and tetracycline and susceptibility to ertapenem, enrofloxacin, cefotaxime, trimethoprim-sulphamethoxazole, florfenicol, apramycin and ceftiofur. All nine representative *E. coli* isolates tested negative for the presence of *E. coli* K99 by ELISA.

### Whole-genome sequencing

#### Strain typing and virulence factors.

The nine representative isolates were submitted for whole-genome sequencing (Table 1 and S1 in S1 File). Genome analysis found that all nine isolates belonged to serotype O78, H9 and multilocus sequence type 23 (ST23). Virulence genes *cma, cvaC, fyuA, gad, irp2, lpfA, mchF, sitA, terC, iss, iroN, hlyF* and *ompT* were identified in all isolates. The latter four genes are associated with APEC strains, and are associated with serum survival, haemolysin production, cytotoxic protein production and iron transport. All isolates lacked shiga toxin genes and a key gene associated with APEC – *iutA* – which is responsible for the expression of an aerobactin receptor. The *fimH* type in these strains contains a single non-synonymous mutation in comparison to the previously described *fimH*-1253 variant, which results in G66S change. As such, this is a novel variant designated *fimH*-1571 and entered onto the Center for Genomic Epidemiology FimTyper database.

#### Antimicrobial resistance genes.

Four antimicrobial resistance genes were detected in all nine genome-sequenced isolates. *tet*(B) confers resistance to tetracyclines, and *blaTEM-1B* confers resistance to some β-lactam antibiotics when over-produced (i.e., ampicillin, cephalothin, piperacillin, amoxicillin and ticarcillin). Two genes were detected that confer resistance to streptomycin – *aph*(3”)-*Ib* and *aph*(6)-*Id*.

### Phylogenetic analysis

A core genome SNP phylogeny was constructed to establish the relatedness to other ST23 isolates (Table S1 in S1 File) and is visualised in Fig 1A and B. All nine study isolates were closely-related, separated by a total of only seven polymorphic positions and a pairwise distance of 0–5 SNPs. Five isolates, two of the five Unit A isolates, C468866 and C468904, and all three isolates from Unit B (C469009, C469142/1 and C469142/2) were indistinguishable. The remaining three isolates from Unit A, C468983, C469032 and C469075, each had a single unique SNP with the single Unit C isolate, C470773, the most distinct with four unique SNPs and therefore distinguished by four or five SNP from the other eight isolates. From the isolates selected from Enterobase, those most closely related to the study *E. coli* all came from poultry (6 isolates from chicken (*Gallus gallus domesticus*) and 3 isolates from turkey (*Meleagris gallopavo*)): one from the UK, two from Italy and five from the USA. The closest isolate in the analysis, an Italian turkey isolate, SAP0709 (accession SRR8923934), was separated from closest bovine study isolates by 54 SNPs.

**Fig 1:**
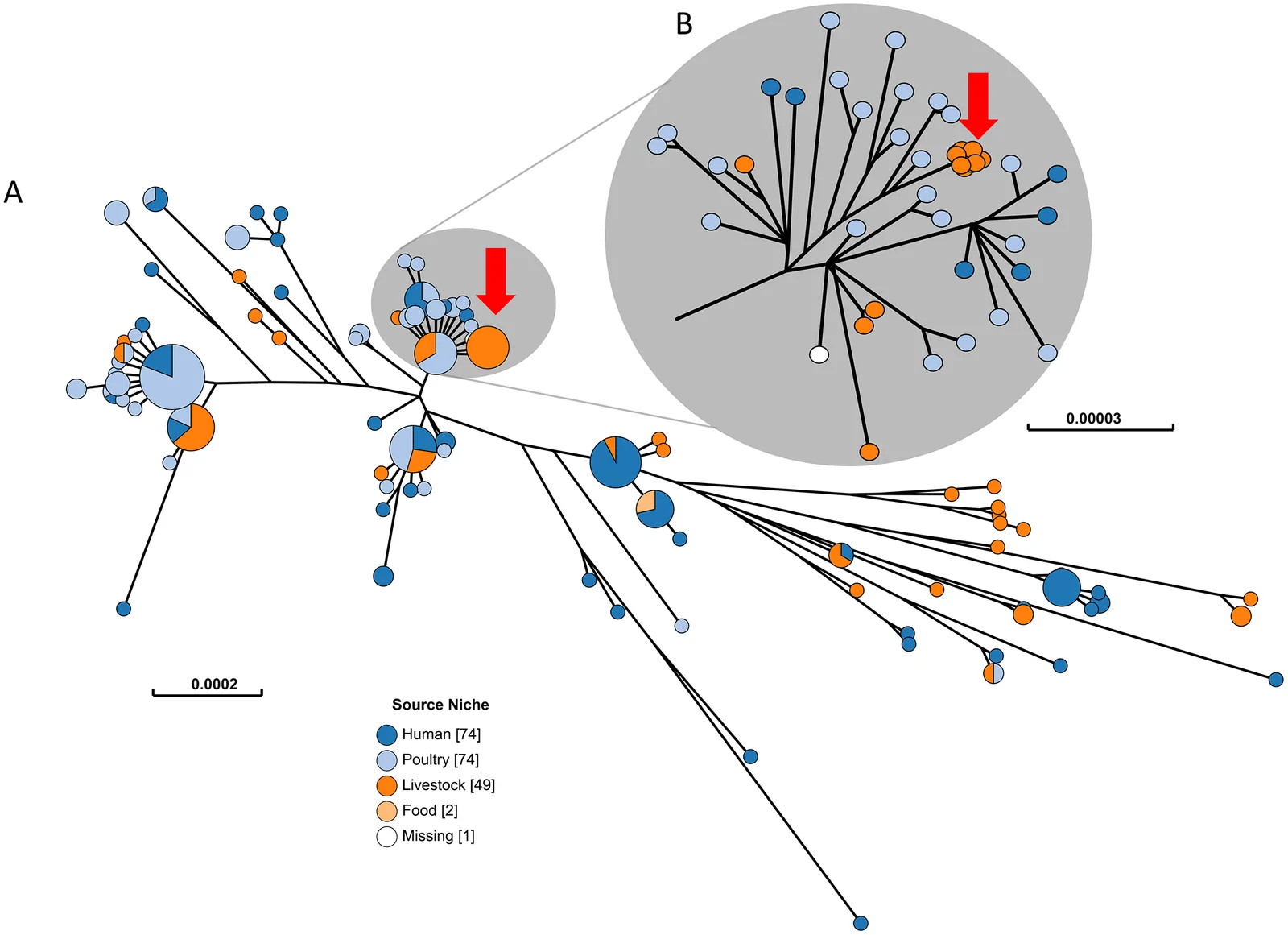
Core genome SNP-based phylogeny of 200 ST23 *E*. *coli.* The grey shading indicates the clade containing the nine calf colisepticaemia isolates, shown in the overall phylogeny and in a magnified insert. The red arrows specifically indicates the nine calf colisepticaemia isolates genome sequenced in this study. The scale bars represent nucleotide substitutions per site.

## Discussion

We describe three farm-level incidences of colisepticaemia, which presented in 3–6 month-old calves. Routine bacteriological culture detected *E. coli* in pure or dominant growth, in most cases heavy growth, in all sampled tissues, which is an unusual clinical presentation in calves of this age, colisepticaemia typically presenting in neonatal calves in association with hypogammaglobulinaemia [3–5]. In all cases, there appeared to be no obvious predisposing factors leading to colisepticaemia development. In particular, there were no significant gastrointestinal lesions or evidence of immunosuppression, specifically, due to exposure to bovine viral diarrhoea virus were found. Consequently, nine representative bacterial isolates, one from each affected calf, were subject to further analysis by whole-genome sequencing for strain typing, assessment of virulence and antimicrobial resistance determinants, and phylogenetic relationships with other sequenced *E. coli* strains of human and animal origin. The selection of nine colonies for sequencing is a limitation of the study as diversity in the disseminated *E. coli* population may have been missed.

### Prevalence of *E.coli* O78 in non-avian species

All nine genome-sequenced isolates studied belonged to serotype O78, which are frequent members of the intestinal microbiota in both sheep and cattle. *E. coli* O78 strains have predominantly been implicated in avian disease [19–24] but there are also studies which describe disease in both ovine [25] and bovine [2,26,27] hosts. Kjelstrup *et al*. (2013) described an outbreak of septicaemia in neonatal lambs caused by *E. coli* O78 [25] and the farm had no known previous history of *E. coli* septicaemia. A further study revealed that 5% of *E*. *coli* isolates from calves in England and Wales with septicaemia were typed as O78 [27]. However, these studies describe *E. coli* O78 infection in newly-weaned calves, rather than calves that are several months old as described here. This may be due to *E. coli* not being considered as an aetiological agent this far forward from weaning, physiological or immunological challenges in the studied cattle, enhanced virulence in the studied strains, or indeed a combination of these factors.

### Antimicrobial resistance

The studied *E. coli* O78 strains are multi-drug resistant, showing phenotypic resistance to ampicillin, amoxicillin-clavulanic acid, streptomycin, and tetracycline. Therefore, fulfilling the MDR criteria of non-susceptibility to at least one agent in three or more antimicrobial classes (e.g., β-lactams, aminoglycosides and tetracyclines). This agrees with genotypic predictions, with genes linked to tetracycline, *tet*(B), ampicillin and amoxicillin-clavulanic acid *bla*_TEM-1B_ and streptomycin, *aph*(3”)*-Ib* and *aph*(6)-Id being detected. While the TEM-1B β-lactamase enzyme is biochemically susceptible to inhibition by clavulanic acid, its increased production can lead to inhibitor saturation resulting in such strains showing reduced susceptibility or resistance to amoxicillin-clavulanic acid [28–30]. This may therefore account for the amoxicillin-clavulanic acid resistance observed here, although the potential increased production of TEM-1B has not been experimentally confirmed nor its genetic basis determined.

In a recent study, AST data was made available for 8713 *E. coli* isolates (including *E.coli* O78 strains) from diseased or deceased diarrhoeic calves between 2015 and 2019 [26]. High rates of multi-drug resistant (84.2%) and extensively drug-resistant (15.8%) isolates were characterised, with notable resistance levels against tetracycline, ampicillin, amoxicillin-clavulanic acid and streptomycin, reflecting our data. A further study (also including O78 strains) also confirmed a high prevalence of resistance against these aforementioned antibiotics [31], highlighting a core pattern of multi-drug resistance and the importance of continued AMR surveillance and antibiotic stewardship.

### Overview of virulence factors

Here, whole-genome sequencing data was utilised to provide insight into the carriage of virulence factors in the study strains. Each of the nine genome sequenced strains carried the same series of virulence genes described here. Firstly, we identified *iss, iroN, hlyF* and *ompT* – four of the five core genes linked with APEC strains [32]. The *sitABCD* operon was also detected, which is linked to resistance to oxidative stress and hydrogen peroxide, and is also highly associated with APEC and human extraintestinal pathogenic *E. coli*, in comparison to commensal and diarrhoeagenic *E. coli* isolates [33].

The fifth virulence gene typical in APEC strains, *iutA*, was not detected in any of the nine strains. This may simply be an artefact due to the limitations of using short-read sequencing, however, a previous study has shown that the *iutA* gene was found in significantly lower frequency in *E. coli* detected in humans with a more severe bacteraemia [34]. The authors hypothesised that *iutA*-expressing isolates may have been detected and eliminated by the immune system before translocation to the blood. Other studies have described the importance of the *iutA* gene for virulence in poultry [35], with a 82.2% prevalence in well studied APEC strains [36].

*iss* has been previously associated with increased serum survival and persistence [37], and septicaemia in the absence of toxin production [25]. We did not detect any known toxin-associated genes in our strains, but it has been shown that toxins are potentially not crucial virulence factors in septicaemic *E. coli* strains [25,38].

*E. coli* strains causing septicaemia are known to carry a range of genes encoding iron uptake systems [39]. Previously, *iroN* expression was increased by incubating *E. coli* strains in human urine or blood and has been significantly linked to *E. coli* virulence in the context of a mouse urinary tract infection model [39,40]. Ewers *et al*., found an abundance of iron acquisition genes among their studied *E. coli* O78 strains, and stated that iron scavenging contributes to the virulence of these organisms, particularly in the context of extraintestinal diseases [2]. Our data reflect these findings, with several genes linked to iron acquisition being present in the nine genome-sequenced isolates.

The *irp2-fyuA* gene cluster has been shown to be key in the synthesis of a High Pathogenicity Island (HPI), with a knock-out of these genes being linked to weakened APEC adhesion and pathogenicity in a chick model [41]. Interestingly, in a screen of *E. coli* O78 isolates from a broad range of hosts, these genes were absent in all sheep isolates [42]. Further to the *irp2-fyuA* gene cluster, we also identified *hlyF*, which plays a key role in extraintestinal APEC infection in a chicken model of colibacillosis [43].

Previously, other authors described a high mortality sepsis in newborn lambs and goats caused by O78 strains, which carried a plasmid encoding the production of K99 fimbriae [44]. Due to this link, we investigated whether our isolates expressed K99 fimbriae using both WGS data and a K99-specific ELISA test, neither of which detected K99. However, we identified a novel *fimH* variant which encodes the tip fibrillum of Type 1 fimbriae and which mediates binding to mannose on host cell surface glycoproteins. Present in all nine study isolates, this novel variant, designated *fimH*-1571, has a single amino acid mutation, G66S, when compared to the previously described *fimH*-1253 variant. Residue 66 of FimH lies within the lectin domain near to the mannose-binding pocket, with the G66S mutation shown to enhance FimH binding to monomannose and to display less dependence on fluid shear to activate high-affinity binding [45,46]. The G66S mutation is also associated with *E*. *coli* isolates from Crohn’s Disease patients and the more-virulent-associated B2/D phylogroups [47], suggesting it represents a pathoadaptive mutation associated with enhanced adhesion/virulence. In the case of colisepticaemia, this FimH mutation hypothetically could contribute to disease pathogenesis by enhancing bacterial adhesion to host tissues, including the large intestine, and potentially increasing the opportunity for invasion into the bloodstream. Although any contribution to disease pathogenesis would require to be confirmed experimentally.

*E. coli* O78 have been known to cause disease in a wide range of hosts, including ruminants [2,26,48,49]. *E. coli* O78 isolates from diarrhoeal cases in neonatal calves and lambs have previously shown high pathogenicity *in vitro* [2,50], and has been shown to cause a severe disease in young ruminants [2,25]. Interestingly, several authors have described that fewer virulence factors were found in highly pathogenic *E.coli* O78 strains from ruminants, in comparison to *E.coli* O78 strain K46 isolates from healthy lambs [25] or other non-O78 strains [48,49]. In the former study, genetic characterisation could not explain the high virulence of this studied pathogenic strain.

#### Phylogenetics.

All nine genome sequenced study isolates, despite being collected from three different premises, were closely-related to each other with five being indistinguishable, which include isolates found on Units A and B. Although not demonstrated here, this suggests an epidemiological link between the isolates and premises, either direct transmission between them or from a common source. The work highlights the utility of genome sequencing to identify otherwise cryptic relationships. Based on the core genome SNP phylogeny, the study isolates were related to those from poultry, consistent with their APEC-like genotype. Although a source tracking investigation was not possible no proximal poultry source was apparent at any of the three premises. The clade, (Fig 1B) with the study and poultry isolates, also contained human isolates, pointing to a potential zoonotic risk which may merit further examination.

## Conclusion

The *E. coli* O78 isolates, responsible for septicaemia and meningitis in three separate dairy units, in three to six-month-old calves appear to be part of a distinct lineage in terms of genetic background, host range and geography. It has been shown that *E. coli* O78 isolates have worldwide clonal relatedness across human, bovine, ovine, porcine and avian sources [44,49], demonstrating a broad host range and zoonotic potential. This work highlights the strength of WGS to establish phylogenetic relatedness of important pathogenic isolates and identify otherwise cryptic associations. Further work is now required to better understand the unusual and virulent clinical presentation of these cases.

## Supporting information

S1 File This contains S1 Table, AST interpretive criteria used in study and S2 Table, Metadata for isolates included in Fig 1 as downloaded from EnteroBase. (XLSX)
